# RETRACTION: Efficacy of the Combination of Chinese Herbal Medicine and Negative Pressure Wound Therapy in the Treatment of Patients With Diabetic Foot Ulcer: A Meta‐Analysis

**DOI:** 10.1111/iwj.70327

**Published:** 2025-03-06

## Abstract

**RETRACTION:** LiJ. , WangH.‐Y. , YangY.‐F. , WangA.‐N. , ShiY. , and CuiT.‐B. , “Efficacy of the Combination of Chinese Herbal Medicine and Negative Pressure Wound Therapy in the Treatment of Patients With Diabetic Foot Ulcer: A Meta‐Analysis,” International Wound Journal 21, no. 4 (2024): e14536, 10.1111/iwj.14536.38069543 PMC10961048

The above article, published online on 08 December 2023, in Wiley Online Library (http://onlinelibrary.wiley.com/), has been retracted by agreement between the journal Editor in Chief, Professor Keith Harding; and John Wiley & Sons Ltd. Following an investigation by the publisher, all parties have concluded that this article was accepted solely on the basis of a compromised peer review process. The editors have therefore decided to retract the article. The authors did not respond to our notice regarding the retraction.

**RETRACTION:** J. Li , H.‐Y. Wang , Y.‐F. Yang , A.‐N. Wang , Y. Shi , and T.‐B. Cui , “Efficacy of the Combination of Chinese Herbal Medicine and Negative Pressure Wound Therapy in the Treatment of Patients With Diabetic Foot Ulcer: A Meta‐Analysis,” International Wound Journal 21, no. 4 (2024): e14536, 10.1111/iwj.14536.38069543 PMC10961048

The above article, published online on 08 December 2023, in Wiley Online Library (http://onlinelibrary.wiley.com/), has been retracted by agreement between the journal Editor in Chief, Professor Keith Harding; and John Wiley & Sons Ltd. Following an investigation by the publisher, all parties have concluded that this article was accepted solely on the basis of a compromised peer review process. The editors have therefore decided to retract the article. The authors did not respond to our notice regarding the retraction.

